# In-vivo expressed *Mycobacterium tuberculosis* antigens recognised in three mouse strains after infection and BCG vaccination

**DOI:** 10.1038/s41541-021-00343-2

**Published:** 2021-06-03

**Authors:** Mariateresa Coppola, Fabienne Jurion, Susan J. F. van den Eeden, Hermann Giresse Tima, Kees L. M. C. Franken, Annemieke Geluk, Marta Romano, Tom H. M. Ottenhoff

**Affiliations:** 1 Department of Infectious Diseases, Leiden University Medical Center, Leiden, The Netherlands; 2 In vivo models unit, Immune Response Service, Infectious Diseases in Humans Scientific Directorate, Sciensano, Belgium

**Keywords:** Tuberculosis, Protein vaccines

## Abstract

Novel tuberculosis (TB)-vaccines preferably should (i) boost host immune responses induced by previous BCG vaccination and (ii) be directed against *Mycobacterium tuberculosis* (*Mtb*) proteins expressed throughout the *Mtb* infection-cycle. Human *Mtb* antigen-discovery screens identified antigens encoded by *Mtb*-genes highly expressed during in vivo murine infection (IVE-TB antigens). To translate these findings towards animal models, we determined which IVE-TB-antigens are recognised by T-cells following *Mtb* challenge or BCG vaccination in three different mouse strains. Eleven *Mtb*-antigens were recognised across TB-resistant and susceptible mice. Confirming previous human data, several *Mtb*-antigens induced cytokines other than IFN-γ. Pulmonary cells from susceptible C3HeB/FeJ mice produced less TNF-α, agreeing with the TB-susceptibility phenotype. In addition, responses to several antigens were induced by BCG in C3HeB/FeJ mice, offering potential for boosting. Thus, recognition of promising *Mtb*-antigens identified in humans validates across multiple mouse TB-infection models with widely differing TB-susceptibilities. This offers translational tools to evaluate IVE-TB-antigens as diagnostic and vaccine antigens.

## Introduction

Tuberculosis (TB) remains the most widespread and deadly infectious disease from a single bacterial pathogen worldwide^1^. The transmission of its causative agent, *Mycobacterium tuberculosis* (*Mtb*), cannot be sufficiently controlled -in adolescents and adults- by childhood vaccination with Bacillus Calmette-Guérin (BCG), the only currently licensed TB vaccine^2^. Therefore, numerous efforts in the last decades have attempted to develop better TB vaccines^3^. Two recent and independent clinical efficacy trials showed that BCG re-vaccination led to a decrease in sustained *Mtb* infection, and that multiple injections of an adjuvanted TB subunit vaccine, M72/AS01E, were able to prevent development of TB in latently *Mtb* infected individuals^4,5^. Although the efficacy of M72/AS01E still needs to be corroborated in larger cohorts and across different geographical areas^6^, this finding underscores the clear potential of using selected *Mtb* antigens as BCG booster vaccines^2^. However, immune correlates of TB protection remain unknown, posing a challenge to the identification of such protective *Mtb* antigens for vaccine design^7^.

In recent years, we have identified a class of *Mtb* antigens, named IVE-TB antigens, encoded by *Mtb* genes that are highly and consistently expressed in the lung of TB susceptible (C3HeB/FeJ) as well as TB resistant (C57BL/6J) mice following aerosol *Mtb* (Erdman) challenge^8,9^. Because these *Mtb* genes are highly expressed in vivo, the encoded IVE-TB proteins represent an interesting set of candidate antigens for several reasons. First of all, they are preserved among at least 219 *Mtb* clinical isolates and other pathogenic mycobacteria, thus offering the potential to elicit immune responses against multiple human virulent *Mtb* strains. Secondly, they share high homology with BCG and are thus likely implementable in booster vaccine strategies. Additionally, they contain multiple predicted HLA-Ia and HLA-II binding motifs covering 85% of the human population. Lastly but most importantly, many IVE-TB antigens were found to be recognised by immune cells of latently *Mtb* infected (LTBI) subjects and TB patients^9,10^. Interestingly, these T cells were capable of producing multiple cytokines, in addition to or even in the absence of IFN-γ, a cytokine known to be necessary but not sufficient in conferring protection against TB^9,10^.

Due to the absence of human *Mtb* challenge models and validated correlates of protection, promising candidate vaccines are commonly tested in mice before being evaluated in larger animal models and eventually clinical trials^11^. Many *Mtb* antigens, such as those included in the most advanced candidates in the TB vaccine pipeline^12–16^, have been screened previously in C57BL/6 and BALB/c^17^. Both strains (particularly C57BL/6) are relatively resistant to TB and do not display the full spectrum of human TB pathology, such as the development of centrally necrotic lesions^18^. By contrast, the less frequently used C3HeB/FeJ strain (also known as the Kramnik model) presents with necrotic lesions and lung cavitation resembling those found in human TB^19^.

In order to translate our previous findings from human cohorts towards highly controlled experimental in vivo TB models in which protective efficacy of candidate vaccines can be explored, we aimed to: (I) evaluate promising IVE-TB antigens across different strains of *Mtb* infected mice, including not only TB resistant (C57BL/6, BALB/c), but also TB susceptible (C3HeB/FeJ) strains, reasoning that the recognition of antigens in mice with different genetic backgrounds, and widely different TB susceptibilities, would better reflect the TB heterogeneity found in the human population; (II) study whether IVE-TB antigens induce IFN-γ as well as non-IFN-γ cytokine responses (TNF-α and IL-17) also in mice and in different organs (i.e., spleen, lung and mediastinal draining lymph nodes), to enable identification of those antigens that, though recognised in all mouse strains, would induce different cytokines profiles in resistant and susceptible mice. Upon asserting that an antigen is recognised in the susceptible strain, the cytokine and cellular responses can then be redirected towards a protective response (e.g., the response found in the resistant strains) by vaccination using appropriate adjuvants. (III) To determine which IVE-TB antigens recognised during *Mtb* infection are also recognised following vaccination with BCG, with the rationale to discriminate between highly specific *Mtb* antigens that could be useful candidates for TB diagnostics in areas with standard neonatal BCG vaccination programs, versus *Mtb*/BCG shared antigens that can be used as BCG booster vaccines; (IV) and validate findings in a multi-laboratory approach across two different laboratories and experimental settings to strengthen confidence in the results, especially in light of the large heterogeneity distinctive of human TB. The latter conforms to recent recommendations, which encourage researchers to perform pre-clinical discovery screenings in different mouse strains, routes of infection, *Mtb* strains and laboratories, before moving to more costly and resource demanding animal and human studies^17^.

We find 19 *Mtb* antigens that are significantly recognised by cells isolated from different murine tissues, presenting a wide array of immune responses with respect to the cytokines produced and the three mouse genetic backgrounds investigated. Of note, the recognition of 11 of these antigens was corroborated in an independent setting. Thus, our multi-laboratory approach identified promising *Mtb* antigenic targets for potential application as tools in TB control.

## Results

### IVE-TB antigen recognition in multiple organs of three genetically different mouse strains by cells producing different cytokines

**Fig. 1:**
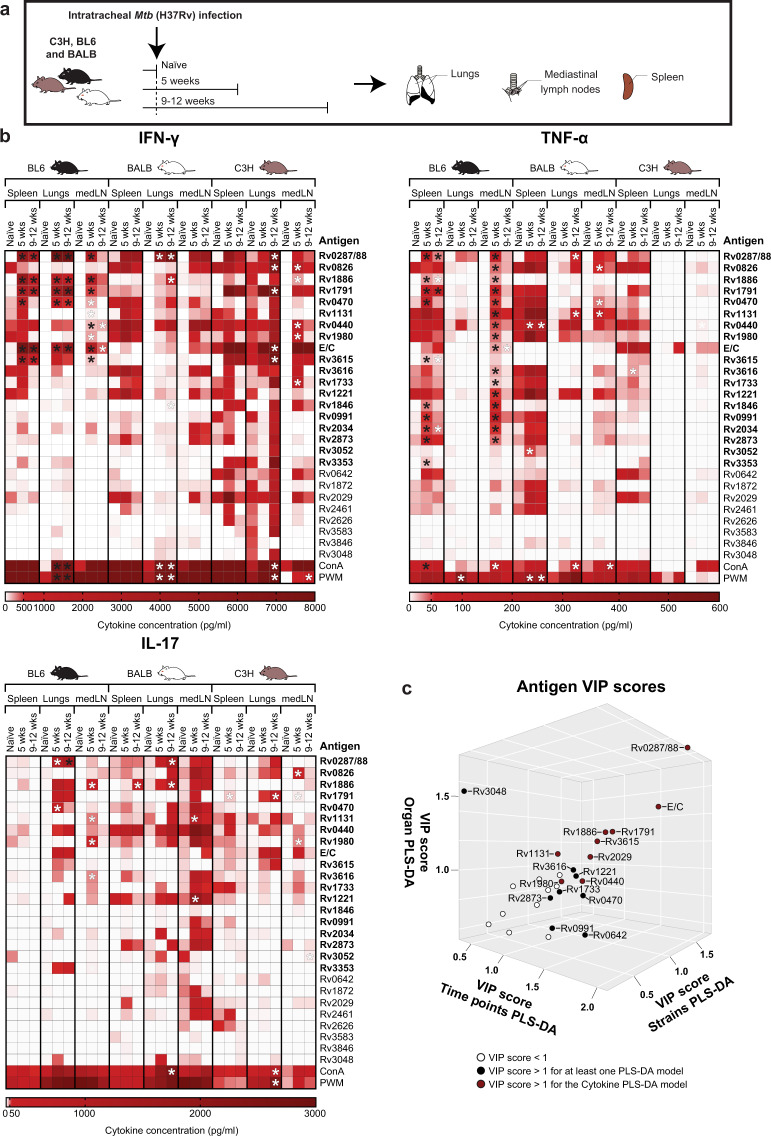
Most IVE-TB antigens are recognised by cells from *Mtb* infected mice with differences among mouse strains, organs and cytokines. **a** C57BL/6 (BL6), BALB/c (BALB) and C3HeB/FeJ (C3H) mice were challenged with 1 × 10^4^ CFU *Mtb*-lux strain intratracheally and killed at an early (5 weeks) or later (9–12 weeks) stage of *Mtb* infection. Uninfected naïve control mice were killed and tested in parallel. Pooled cells from lungs, mediastinal lymph nodes (medLN) or spleens were stimulated with single or fusion *Mtb* antigens or with positive control stimuli ConA and PWM. The concentrations of IFN-γ, TNF-α and IL-17 were measured in the cell supernatants after 3 days of stimulation and corrected for background. **b** Heatmaps of the specific *Mtb* antigen responses for each cytokine are shown. Colour gradients indicate the cytokine concentration, while asterisks indicate significant differences between the naïve and the *Mtb* challenged groups. White *p-value < 0.05, computed with Kruskal–Wallis test; black asterisk marks differences that remained significant after multiple test correction (*q*-value < 0.1, i.e., FDR-adjusted *p*-values of Mann–Whitney.test). **c** 3D plot of the Variable Importance in Projection (VIP) scores obtained from each PLS-DA model computed on the basis of the following known classes: (i) time points (i.e., naïve mice vs. mice killed at early or late point after *Mtb* infection); (ii) mouse strains (i.e., C57BL/6 (BL6), BALB/c (BALB) and C3HeB/FeJ (C3H) mice); (iii) organs (i.e., lungs, mediastinal lymph nodes or spleens); (iv) cytokines (i.e., IFN-γ, TNF-α and IL-17). Antigens with a VIP score >1 were considered to have an above average influence on the model and are depicted by solid dots. Antigens with a VIP score >1 for the cytokine PLS-DA model are depicted by solid red dots. Antigens with a VIP score <1 are depicted by empty dots and their names are omitted in the 3D plot.

Promising TB candidate vaccine antigens first need to be validated in small animal models before they can be selected for evaluation in larger animal models such as non-human primates, and eventually for testing in human clinical trials^11^. Here, we aimed to verify which IVE-TB antigens, previously shown to be recognised by T cells from TB patients and LTBI subjects^9,10^, were consistently and broadly recognised during the course of murine *Mtb* infection in different genetic backgrounds, given the fact that human populations are genetically highly diverse. To include different genetic backgrounds with widely diverse TB-susceptibility phenotypes, we selected C57BL/6, BALB/c and C3HeB/FeJ mice and challenged these with *Mtb*^20,21^. Different cell pools from each organ (spleens, lungs and mediastinal lymph nodes (medLN)), strain and time-points (early (5 weeks) or later (9–12 weeks) stage of *Mtb* infection) were tested in parallel by stimulation with single or fusion IVE-TB antigens (*n* = 22) (Table 1) as well as with positive controls (ConA and PWM) (Fig. 1a, Supplementary Data 1). Besides IVE-TB antigens, also several other, stage specific- and secreted-*Mtb* antigens (*n* = 5) were included in the screening since several studies previously described these as promising (diagnostic) TB biomarkers or as putative antigens for TB vaccination^22–25^ (Table 1). The concentrations of IFN-γ, TNF-α and IL-17 were measured in the cell supernatants after 3 days of stimulation. Cytokine levels in response to each *Mtb* antigen were compared between naïve mice and *Mtb* challenged mice for each organ and mouse strain, after subtracting background concentrations from unstimulated negative control samples (Supplementary Data 1).

**Table 1:** List of *Mtb* antigens included in this study.

List	Rv number	Gene name	Category	Reference(s)
1	**Rv0287/Rv0288**	EsxG/EsxH	IVE-TB	Coppola 2016
2	**Rv0440**	GroEL2	IVE-TB	Coppola 2016
3	Rv0470c	PcaA	IVE-TB	Coppola 2016
4	Rv0642c	MmaA4	IVE-TB	Coppola 2016
5	Rv0826	Conserved hypothetical protein	IVE-TB/ latency antigen	Coppola 2016; Rustad 2008
6	Rv0991	Conserved serine rich protein	IVE-TB	Coppola 2016
7	Rv1131	PrpC	IVE-TB	Coppola 2016
8	Rv1221	SigE	IVE-TB	Coppola 2016
9	**Rv1791**	PE19	IVE-TB	Coppola 2016
10	Rv1846	BlaI	IVE-TB	Coppola 2016
11	Rv1872	IldD2	IVE-TB	Coppola 2016
12	**Rv1980c**	Mpt64	IVE-TB	Coppola 2016
13	Rv2461	ClpP1	IVE-TB	Coppola 2016
14	**Rv2626**	Hrp1	IVE-TB/latency antigen	Coppola 2016; Commandeur 2011; Serra-Vidal 2014
15	**Rv2873**	Mpt83	IVE-TB	Coppola 2016
16	Rv3048c	R1F protein	IVE-TB	Coppola 2016
17	Rv3052	FadB4	IVE-TB	Coppola 2016
18	Rv3583c	Possible transcription factor	IVE-TB	Coppola 2016
19	**Rv3615**	EspC	IVE-TB	Coppola 2016
20	Rv3616*	EspA	IVE-TB	Coppola 2016
21	Rv3846	SodA	IVE-TB	Coppola 2016
22	**Rv3874/Rv3875**	ESAT6/CFP10 (E/C)	IVE-TB/secreted antigens	Van Pinxteren 2000; Coppola 2016
23	Rv1733c	Rv1733c	latency antigen	Commandeur 2011; Serra-Vidal 2014
24	Rv2034	Rv2034	IVE-TB stage specific	Commanduer 2013
25	Rv3353c	Rv3353c	IVE-TB stage specific	Commanduer 2013
26	Rv2029c	Rv2029c	latency antigen	Commandeur 2011; Serra-Vidal 2014
27	Rv1886c	Ag85B	secreted antigens	Babaki 2017

Previously described immunodominant *Mtb* specific antigens are shown in bold font. Gene names were reported according to their annotations in the Mycobrowser data repository (https://mycobrowser.epfl.ch/).

*Mtb* mycobacterium tuberculosis, *IVE-TB* in vivo expressed Mtb antigens.

^a^patent WO 2014063704 A2.

Several trends emerged when grouping the cytokine responses against the 27 *Mtb* antigens included in this study (Supplementary Fig. 1). High TNF-α responses were found to the majority of the selected *Mtb* antigens, most predominantly in the spleen, in all three mouse strains. Secondly, the cells analysed from the C3HeB/FeJ mice showed an overall reduced ability to produce TNF-α against *Mtb* antigens, especially cells from the medLN and lungs whose TNF-α response was low or absent even after mitogen stimulation (Fig. 1b and Supplementary Fig. 1). Antigen-specific IFN-γ and IL-17 responses were more diversely distributed and varied strongly between organs and mouse strains (Fig. 1b). Interestingly, especially in BALB/c and C57BL/6 mice, significant IL-17 responses were mainly induced in cells isolated from the lungs and medLN, while IL-17 responses were almost absent among the splenocytes (Supplementary Fig. 1). For splenocytes and medLN, the highest peak of cytokine production was mostly observed at an early stage of *Mtb* infection, while for lung cells this was observed at the later stage (Supplementary Fig. 1). IFN-γ responses tended to be highest in C3HeB/FeJ mice, the most TB susceptible strain, confirming that like in humans IFN-γ in itself is not sufficient for protective immunity.

Among the *Mtb* antigens that induced a significant specific increase in cytokine production, the highest number was recognised by medLN cells from C57BL/6 mice (18/27 antigens recognised), followed by C3HeB/FeJ (7/27 antigens recognised) and BALB/c (4/27 antigens recognised) mice (Fig. 1b).

From these antigens, only one was recognised by all mouse strains (Rv0826), five by C57BL/6 and C3HeB/FeJ (Rv1886, Rv0440, Rv1980, Rv1791 and Rv1733), and 3 by C57BL/6 and BALB/c mice (Rv1131, Rv1221 and Rv0470). In the C57BL/6 mice and, to a lesser extent, in the other two mouse strains, medLN cells produced multiple cytokines in response to single *Mtb* antigens. The different combinations found were: IFN-γ, TNF-α and IL-17 against 3 antigens (in C57BL/6 mice: Rv1886, Rv1131 and Rv1980); IFN-γ and TNF-α against 5 antigens (in C57BL/6 mice: Rv0287/88, Rv0470, Rv1791, ESAT6/CFP10 and Rv0440. For Rv0440, the same cytokine profile was found in C3HeB/FeJ mice); IFN-γ and IL-17 against two antigens (in C3HeB/FeJ mice: Rv0826 and Rv1980); TNF-α and IL-17 against 2 antigens (in C57BL/6 mice: Rv3616; in BALB/c mice: Rv1131).

Antigen-specific splenocyte responses to *Mtb* antigens were mainly detected – as measured by either one or more of the three cytokines evaluated – in C57BL/6 mice (11 antigens recognised) and to a lesser extent in BALB/c (3 antigens recognised) and C3HeB/FeJ (2 antigens recognised) mice. Among these, only the recognition of one antigen, Rv1886, was shared between C57BL/6 and BALB/c mice, and one other (Rv1791) between C57BL/6 and C3HeB/FeJ. Only splenocytes from C57BL/6 mice produced multiple cytokines, i.e., IFN-γ and TNF-α, against 5 antigens (Rv1886, Rv0287/88, Rv0470, Rv1791 and Rv3615).

Lung cells consistently recognised fewer antigens than cells isolated from other tissues (Fig. 1c). However, some of these antigens were recognised by lung cells of different mouse strains (Rv0287/88 was recognised by all mouse strains; Rv1886 by C57BL/6 and BALB/c; ESAT6/CFP10 and Rv1791 by C57BL/6 and C3HeB/FeJ). Significant levels of IFN-γ and IL-17 were secreted by lung cells in response to 4 antigens (in C57BL/6 mice: Rv0287/88 and Rv0470; in BALB/c mice: Rv1886; and in C3HeB/FeJ mice: Rv1791). Only against one antigen (Rv0287/88) a combined IFN-γ, TNF-α and IL-17 response was found in BALB/c mice.

**Fig. 2:**
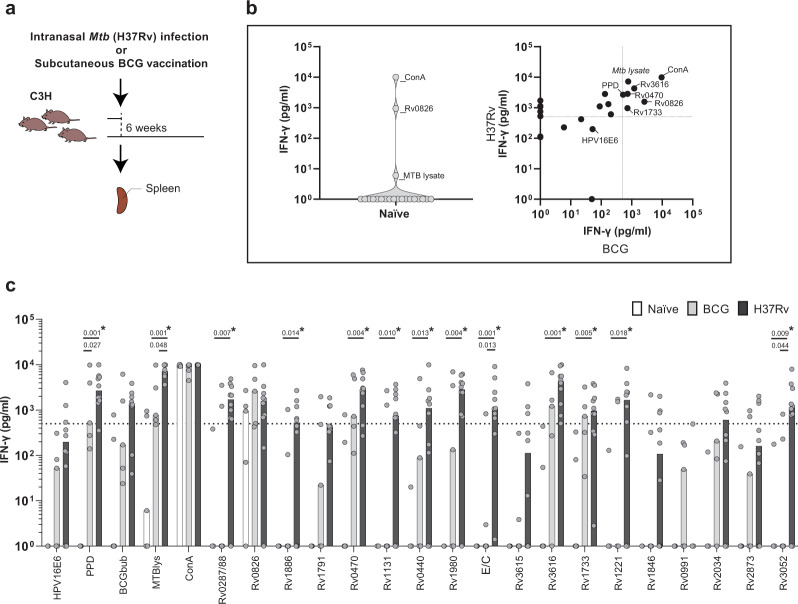
IVE-TB antigen-specific responses after *Mtb* infection and BCG vaccination in C3HeB/FeJ (C3H) mice. **a** C3HeB/FeJ mice were either challenged intranasally with *H37Rv-Mtb* strain (10^5^ CFU) (10 mice), vaccinated subcutaneously with BCG (5 mice) or left untreated (5 mice). After 6 weeks, splenocytes were harvested and stimulated per mouse for 6 days with a set of eighteen *Mtb* antigens or positive controls (ConA, PPD, bead disrupted BCG (BCG bub) and *Mtb* lysate), or negative control (HPV16E6 recombinant protein). The IFN-γ concentrations detected in the unstimulated samples were subtracted from the stimulated samples for each condition within each mouse. **b** IFN-γ production after antigen stimulation in splenocytes from naïve unimmunized mice (left panel), and BCG immunised versus *Mtb* infected mice (right panel). Dots display the median IFN-γ production. The (Rv) codes of the antigens are indicated if the median IFN-γ production exceeded 500 pg/ml in both BCG immunised and *Mtb* infected mice. The horizontal dotted line indicates the cut-off at 500 pg/ml, which is twice the median IFN-γ production found in response to the negative control in BCG immunised and *Mtb* infected mice (right panel). **c** IFN-γ responses against *Mtb* antigens in splenocytes from naïve, BCG immunised and *Mtb* infected mice are shown. Dots represent a single mouse (10 mice in the *Mtb* infected group and 5 mice in the naïve and BCG immunised group in total) and bars indicate the median IFN-γ response of each group. The *Mtb* challenged and BCG immunised groups were compared to the naïve group using the Kruskal–Wallis test and a *p*-value < 0.05 was considered significant. Asterisk marks differences that remained significant after multiple test correction (*q*-value < 0.1, i.e., FDR-adjusted *p*-values of Mann–Whitney test).

Most of the differences listed above were no longer significant after the very stringent FDR correction, but importantly this included also responses to controls and to previously described antigens known to be highly immunogenic such as ESAT6/CFP10 (Figs 1b and 2c). Therefore, the results are shown both with and without FDR correction, to avoid the risk of missing biological relevant candidates at this early stage of discovery.

Interestingly, in the resistant mouse strains, there were a few antigens (Rv0470, Rv0826, Rv0991, Rv1131, Rv1221, Rv1733, Rv1846, Rv1980, Rv2034, Rv2873, Rv3052, Rv3353 and Rv3616) that induced statistically significant TNF-α but not IFN-γ responses. Except for Rv1846, this lack of overall significant IFN-γ responses seemed to be the result of the observed heterogeneity in donor responses rather than the complete absence of this cytokine (Supplementary Fig. 3).

To validate the results in a subsequent independent multivariate analytical approach, we used PLS-DA modelling to determine the key antigens that drove the discrimination in cytokine responses between (i) time points (E/C, Rv0642, Rv0287/88, Rv0991, Rv1791, Rv1886, Rv2029 and Rv3616); (II) mouse strains (E/C, Rv0287/88, Rv0440, Rv0470, Rv0642, Rv1221, Rv1791, Rv1886, Rv2029, Rv2873 and Rv3615); (iii) organs (E/C, Rv0287/88, Rv1131, Rv1791, Rv1886, Rv2029, Rv3048, Rv3615 and Rv3616); and (iv) type of cytokine (E/C, Rv0287/88, Rv0440, Rv1131, Rv1791, Rv1886, Rv1980, Rv2029 and Rv3615), based on the Variable Importance in Projection (VIP) values (VIP > 1). The PLS-DA protein clusters (Supplementary Fig. 2a) were very similar to those obtained by unsupervised PCA (Supplementary Fig. 2b) confirming their validity. (Supplementary Data 1 and Fig. 1c). Of note, this independent multivariate approach confirmed 13 of the 19 proteins that were found as being interesting targets in our first analysis.

Overall, nineteen out of 27 selected *Mtb* antigens (Fig. 1c, in bold) elicited significant cytokine production from cells isolated from *Mtb* infected mice compared to those found in uninfected animals. Of note, although as expected these responses were somewhat heterogeneous, 13 antigens appeared to drive discriminatory cytokine responses between the different mouse strains, time points, type of cytokine and tissues analysed in this study. But perhaps most importantly, the results show that human T cell recognition profiles translate well to mouse models, which was a major aim of this study.

### IVE-TB antigen-specific responses after live BCG vaccination

A TB subunit vaccine, in order to be effective, should preferably boost immune responses induced by prior vaccination^26^. BCG, despite its insufficient protective efficacy against adult TB, remains the only licensed vaccine against TB and is highly effective in protecting against severe childhood TB^2^. To evaluate whether immune responses against IVE-TB antigens could be induced by BCG immunisation, and to compare those responses to those induced by *Mtb* infection, C3HeB/FeJ mice were either challenged with *H37Rv-Mtb* strain (10^5^ CFU) (10 mice), vaccinated subcutaneously with BCG (5 mice) or left untreated (5 mice) (Fig. 2a). After 6 weeks, splenocytes were harvested and stimulated for 6 days with a set of 18 *Mtb* antigens selected from our first screen (Fig. 1c). Positive (ConA, PPD, bead disrupted BCG, *Mtb* lysate) and negative (HPV16E6 recombinant protein) controls were also included. The IFN-γ production in response to the different stimuli was measured and analysed, and as above concentrations detected in the unstimulated samples were subtracted from each condition within each mouse. TNF-α and IL-17 responses were only present at low levels in C3H mice in the first experiment (Fig. 1b) and therefore not assessed in this screening. Three antigens (Rv0470, Rv1733 and Rv3616) were strongly recognised by *Mtb* infected as well as BCG vaccinated C3HeB/FeJ mice (i.e.: IFN-γ production >500 pg/ml) (Fig. 2b). Additionally, despite the different experimental setup and laboratory setting, eleven *Mtb* antigens (Rv0287/88, Rv1886, Rv0470, Rv1131, Rv0440, Rv1980, ESAT6/CFP10, Rv3616, Rv1733, Rv1221 and Rv3052) were confirmed to be significantly recognised after *Mtb* challenge in C3HeB/FeJ mice (Fig. 2c). Interestingly, besides ESAT6/CFP10 (E/C), of which peptides are already widely used as TB diagnostic tool in IGRA tests^27^, Rv0287/88, Rv1886, Rv1131, Rv1221, Rv1846 and Rv3052 were the only antigens for which IFN-γ responses differed highly between the *Mtb* infected and the BCG vaccinated group (Fig. 2c). Prior to FDR correction, differences between the *Mtb* infected and the BCG vaccinated group were significant only for E/C (as expected), Rv3052 and *Mtb* lysate.

Collectively, these findings confirm, extend and partially validate the immune recognition of eleven *Mtb* antigens previously identified in humans across three genetically unrelated mouse TB-infection models and two different independent laboratory settings.

## Discussion

An important requirement for novel TB candidate vaccines is that they evoke immunity to *Mtb* proteins that are expressed, processed and presented to the host immune system during infection. Over the last years, we identified multiple *Mtb* proteins recognised by immune blood cells from latently *Mtb* infected individuals and TB patients^3,9^. During the antigen selection process, we selected proteins encoded by genes highly expressed either during the entire course of infection, or during specific stages of in vivo *Mtb* infection^3^. Before entering clinical studies, candidate vaccines need to be evaluated and proven to be immunogenic in animal studies^11^. Therefore, we here examined a selection of *Mtb* antigens identified in our previous in vitro human studies and backtranslated this to three different, genetically and TB-susceptibility diverse mouse TB models. We found that among a set of 27 *Mtb* antigens recognised by human cells, 19 were able to induce the production of at least one of three cytokines (IFN-γ, TNF-α, IL-17) in at least one of the tissues examined in each of the three mouse strains after challenge with *Mtb*. Further confirmation of our previous data in human cohorts was that several *Mtb* antigens induced considerable cytokine responses other than IFN-γ^9,28^. Importantly, and in accordance with current recommendations^17^, the results were partially validated in a second independent laboratory and experimental setting (Supplementary Table 1), showing recognition of 11 *Mtb* antigens in infected C3HeB/FeJ mice. Among those, three antigens (i.e.: Rv0470, Rv1733 and Rv3616) were found to be recognised also after BCG vaccination. The latter finding is essential when considering the design of BCG-booster vaccines against TB^2^.

Overall, in the experiments performed in the first laboratory, we observed highly significant responses to *Mtb* antigens which varied by magnitude, cytokine combinatorial patterns, tissues and mouse strains (Fig. 1). As previously observed in human studies^9^, the differences in the levels and types of cytokines induced by an antigen, or the fact that certain antigens did not elicit detectable immune responses, is not linked to a particular protein function. Interestingly, non-IFN-γ responses were detected in the spleen and the mediastinal draining lymph nodes of the resistant C57BL/6 mouse strain after stimulation with a substantial number of antigens (Rv0470, Rv0826, Rv0991, Rv1131, Rv1221, Rv1733, Rv1846, Rv1980, Rv2034, Rv2873, Rv3353 and Rv3616), which agrees with our previous findings^9^ and also with the recent description of non-canonical IFN-γ-independent immune responses found in the peripheral blood of *Mtb* exposed “resistant” individuals^28^. Cells from C57BL/6 mice recognised significantly more *Mtb* antigens than those from BALB/c and C3HeB/FeJ mice. Although C57BL/6 and BALB/c mice are equally resistant to TB^29^, several genetic determinants might have contributed to the differences found in the antigens recognised. For instance, the adaptive type 1 immune response to pulmonary mycobacterial infection appears later and with lower magnitude in BALB/c compared to C57BL/6 mice^30,31^. In line with our findings, the lower magnitude of IFN-γ and TNF-α production levels in BALB/c mice was already described in splenocytes and lung lymph node cells upon antigen stimulation^31^. Unexpectedly to us, we found that lung and mediastinal lymph nodes cells from C3HeB/FeJ mice failed to produce detectable TNF-α, not only after *Mtb* antigens exposure, but also upon some of the positive mitogen controls. During mycobacterial infection, TNF-α deficient mice develop necrotic granulomas, pathogenic lesions which are also characteristic of the C3HeB/FeJ mice^32,33^. Although cells isolated from lung of C3HeB/FeJ mice can produce TNF-α, they do that to a lesser extent than C57BL/6 mice, potentially explaining why they develop necrotic lesions. The lower TNF-α production in C3HeB/FeJ mice might be caused by a greater and premature accumulation of a functionally exhausted T cell subsets, characterised by a limited capacity to secrete IL-2 and TNF-α^34^. Demonstrating the recognition of Mtb antigens in this TB susceptible strain was particularly important from a corrective vaccine perspective. Redirecting the cellular response in such strains towards a protective response (e.g., that found in the resistant strain) could be achieved by immunising mice with recognised antigens and appropriate adjuvants (e.g. CAF01), as has been done for instance with ESAT6, which is a strongly antigenic protein in active TB patients, but is also a powerful antigen for vaccination when delivered in CAF01^35^.

Only two antigens were consistently recognised by cells isolated from the same tissue across all mouse strains: Rv0287/88 and Rv0826. Rv0287/88 is a heterodimer secreted by the ESX-3 secretion system, implicated in *Mtb* virulence and survival^36,37^. Lung cells from all three mouse models produced IFN-γ when stimulated with Rv0287/88, while TNF-α and IL-17 were secreted concomitantly in BALB/c mice and TNF-α in C57BL/6 mice. In the latter strain, also splenocytes and mediastinal lymph nodes cells responded to Rv0287/88 by producing IFN-γ and TNF-α. These findings validate and extend what has been shown for lung T cells of CB6F1 (BALB/c × C57BL/6) and B6C3F1 (C57BL/6 × C3HeB/FeJ), which are able to recognise Rv0287/88 after 21 days of *Mtb* infection^38^. Although evidence for the antigenicity of Rv0287/88 accumulates, susceptible C3HeB/FeJ mice immunised with BCG did not recognise this fusion protein. This contrasts with another study, performed in a TB-resistant strain, wherein splenocytes of BCG vaccinated mice responded to specific Rv0288 epitopes^39^. Rv0288 constitutes part of the H4:IC31, an adjuvanted polyprotein vaccine that, though immunogenic, failed to boost neonatal BCG vaccination in a recent prevention of (sustained) infection clinical trial^4^. Testing this vaccine in BCG immunised mice with different TB susceptibility phenotypes might provide improved models that are more predictive of humans.

At an early stage of *Mtb* infection, Rv0826 was recognised by medLN cells from all mice, mainly inducing TNF-α in C57BL/6 and BALB/c mice and IFN-γ and IL-17 in C3HeB/FeJ mice. Rv0826 is a conserved protein with unknown function, highly expressed during enduring hypoxia and in extensively drug resistant *Mtb* strains^40,41^. These characteristics, together with the strong cellular recognition found in humans^42^ and in mice, would make this antigen an attractive target. However, we observed consistent unspecific immune responses against Rv0826 in the splenocytes of all mice, verified in an independent experiment (Fig. 2b), which deserves further investigation. This might be due to cross-reactivity to other microbial antigens, e.g., microbiota derived, but this remains currently speculative.

Besides Rv0287/88 and Rv0826, other antigens were consistently recognised across tissues and mouse strains, and better able to elicit cells producing multiple cytokines (Rv1886, Rv1791, Rv0470, Rv1131, Rv0440, Rv1980) than other antigens (ESAT6/CFP10, Rv3615, Rv3616, Rv1733, Rv1221, Rv1846, Rv0991, Rv2034, Rv2873, Rv3052 and Rv3353). Among those, Rv0470c was particularly interesting because it is an IVE-TB antigen consistently recognised across mice both after *Mtb* challenge and after BCG vaccination, in independent experiments and in different laboratories. Additionally, though recognised in susceptible mice, Rv0470 induced different magnitudes and type of cytokine responses in the resistant strains (Fig. 1b, c). Rv0470c is an enzyme essential for both the synthesis and cyclopropanation of mycolic acids and the cording morphology of both BCG and *Mtb*^43,44^. In addition, Rv0470c prevents phagosome maturation in human monocyte-derived macrophages, while in mice, its deletion alters the persistence and pathology of *Mtb* at late stages of infection^43,45^. Of note, immunisation with BCG_0470 increased the delayed-type hypersensitivity as well as Th1 responses in BCG primed BALB/c mice^46^. All these features, supported by our data in mice and humans^9,10^, point to Rv0470c as an attractive BCG booster vaccine candidate when delivered with a properly selected adjuvant^47^.

Interestingly, Rv0287/88, Rv1886, Rv1131, Rv1221, Rv1846 and Rv3052, though highly homologous to *M. bovis* proteins, were the antigens, besides ESAT6/CFP10, for which IFN-γ response highly differed between *Mtb* infected and BCG vaccinated groups (Fig. 2c)^9^. Comparing the specific responses between TB patients and non *Mtb* exposed BCG vaccinees might reveal the potential of these antigens as adjunct TB diagnostic tools.

In conclusion, this study demonstrates the consistent recognition of several promising IVE-TB *Mtb* antigens among multiple genetically and TB susceptibility diverse *Mtb* infected mouse strains, across various infection models and tissues and time points evaluated, thus offering translational tools to evaluate *Mtb* antigens in appropriate animal models.

## Methods

### Intratracheal *Mtb* infection in C57BL/6, BALB/c and C3HeB/FeJ

C57BL/6, BALB/c and C3HeB/FeJ mice were bred in the animal facilities of Sciensano. Mice or breeding couples were obtained from Janvier Labs (France) for C57BL/6 and BALB/c or from The Jackson Laboratory (USA) for C3HeB/FeJ (JAX stock #000658). Aged-matched female mice 8–15 weeks old at the start of each experiment were used. All *Mtb* infections were performed in a biosafety level 3 (BSL3) facility at Sciensano and all the procedures were performed in accordance with the Belgian legislation and were approved by the ethics committee of Sciensano under the file number 201405–14–01.

Mice were infected intratracheally with a dose of 1 × 10^4^ CFU of virulent and luminescent *M. tuberculosis* H37Rv grown for 2 weeks as a surface pellicle on Sauton medium and stored frozen in aliquots at −80 °C. The *M. tuberculosis* H37Rv strain used is transformed with the reporter plasmid pSMT1, which expresses the *Vibrio harveyi luxAB* genes under the control of the BCG hsp60 promoter^21^. We have previously shown that measuring RLU is an accurate alternative to determining CFU counts^21,48^. Bacterial loads in total lungs of infected mice were verified at the specified time-points in all infected mice for the first screen. For the second and third screens, bacterial loads were measured in the left lung lobe of individual mice of 1 pool/time-point. The other lobes and total lungs of the mice of the other pools were treated with Collagenase/DNAse I for isolation of leucocytes. To measure bacterial loads, the number of bioluminescent organisms [determined as relative light units (RLU)] in lung homogenates was determined by a bioluminescence assay with a Modulus luminometer (Turner Biosystems) and 1% n-decanal in ethanol as a substrate. Data are expressed as mean log10 mRLU values per group (Supplementary Data 1).

### Preparation of cells harvested from intratracheal *Mtb* infected C57BL/6, BALB/c and C3HeB/FeJ mice

Uninfected naïve control mice and infected mice were killed at specified time-points (Supplementary Data 1). Spleens, lungs and mediastinal lymph nodes were removed aseptically and prepared for cell culture. For that purpose, spleens, mediastinal lymph nodes and lungs (first screen, Supplementary Data 1) were gently homogenised in RPMI medium supplemented with penicillin (60 µg/ml) using a Dounce homogenizer. Mediastinal lymph nodes and lungs (first screen, Supplementary Data 1) were passed through a 70-µm nylon cell strainer (Greiner). Lungs (second and third screen, Supplementary Data 1) were fragmented using a scalpel and digested for 1 h at 37 °C and 5% CO_2_ in HBSS (Gibco) supplemented with penicillin (60 µg/ml) and streptomycin (100 µg/ml), 5% of foetal calf serum (FCS), 1 mg/ml of Collagenase from *Clostridium histolyticum* (Sigma-Aldrich, C5138) and 0.1 mg/ml of DNAse I from bovine pancreas (Roche, 11284932001). After incubation, digested lung fragments were gently homogenised in a Dounce homogenizer and passed through a 70-µm nylon cell strainer (Greiner). Single cell solutions of cells isolated from spleen, mediastinal lymph nodes and lungs were washed, counted and cultured in RPMI medium supplemented with 5 × 10^−5^ M 2-mercaptoethanol, penicillin (60 µg/ml) and streptomycin (100 µg/ml), and 10% FCS (number of cells in Supplementary Data 1). Cells isolated from the same organ and mouse strain were pooled in order to have a sufficient number of leucocytes. Pools of cells isolated from 4–13 organs/experimental group were cultured for 72 h with recombinant proteins (10 µg/ml) or RPMI as a negative control (defined as unstimulated samples) or Pokeweed (PWM, 20 µg/ml, Sigma, Cat. N° L9379) or Concanavalin A (ConA, 4 µg/ml, Sigma, Cat. N° C5275) as positive controls. Cell free culture supernatants from at least three separate wells were pooled and supernatants were stored frozen at −20 °C until analysis by enzyme-linked immunosorbent assay (ELISA) was performed.

### Intranasal *Mtb* infection and sub cutaneous BCG vaccination of C3HeB/FeJ

C3HeB/FeJ female mice purchased from the Jackson Laboratory (USA, JAX stock #000658) were used in this study at 6 weeks of age. C3HeB/FeJ mice were infected with 1 × 10^5^ CFU live *Mtb* strain H37Rv from glycerol stocks, stored at −80 °C. Mice were anaesthetised with 2-chloro-2-(difluoromethoxy)-1,1,1-trifluoro-ethane (isofluoran; Pharmachemie BV, The Netherlands) and intranasally (i.n.) infected. Six weeks post *Mtb* challenge, mice were euthanized with CO_2_ and lungs and splenocytes were aseptically removed. Organs were homogenised using 70 µM cell strainers (Corning, U.S.A.) and the amount of *Mtb* bacteria was determined by plating out serial dilutions of the homogenates on 7H11 plates (BD Bioscience, U.S.A), supplemented with BBL Middlebrook OADC enrichment (BD Bioscience, U.S.A) and PANTA (BD Bioscience, U.S.A). Colonies were counted after 3 weeks of incubation at 37 °C (Supplementary Data 2). For BCG immunisation, C3HeB/FeJ mice were injected subcutaneously in the right flank with 10^6^ CFU BCG1331 (SSI, Denmark) from glycerol stocks, stored at −80 °C. Six weeks post BCG vaccination, mice were killed and splenocytes were aseptically removed. Naïve mice were included as control. All mice were daily monitored for ethical requirements, and weighed once a week according to the ethical regulations at the LUMC animal facility (DEC number 11183).

### Recombinant proteins

A total of 27 *Mtb* recombinant proteins, previously identified by different *Mtb* antigen discovery approaches^3^, were tested in this study (Table 1). As described previously^9^, *Mtb* genes as well as the HPV16E6 gene, were amplified by PCR from genomic H37Rv DNA and cloned by Gateway technology (Invitrogen, Carlsbad, CA, USA) in a bacterial expression vector, overexpressed in *Escherichia coli* (E. coli) BL21 (DE3) and purified. Gel electrophoresis and western blotting with an anti-His tag Antibody (Invitrogen) and an anti-E. coli polyclonal antibody (a kind gift of Statens Serum Institute, Copenhagen, Denmark) were used to check the size and purity of the recombinant proteins. Rv0287/88 and Rv3874/75 were produced as fusion proteins to mirror the pairwise dependent secretion pathway by the mycobacterial T7S system. All recombinant proteins here included were previously tested to confirm lack of protein-non-specific T-cell stimulation and cellular toxicity^9^.

### ELISA on cells harvested from intratracheally *Mtb* infected mice

IFN-γ was quantified by sandwich ELISA using purified rat anti-mouse IFN-γ clone R4-6A2 (BD Biosciences, Cat. No 551216) as the capture antibody, biotinylated rat anti-mouse IFN-γ clone XMG1.2 (BD Biosciences, Cat. No 554410) as detection antibody, peroxidase-conjugated streptavidin (Jackson ImmunoResearch, Cat. No 016-030-084) and recombinant mouse IFN-γ protein (R&D Systems, Cat. No 485-MI) as standard. Plates were revealed with O-phenylenediamine dihydrochloride substrate (SIGMAFAST™ OPD, Cat. No P9187), the reaction was stopped with 1 M H_2_SO_4_, and the optical density was read at 490 nm on an Infinite F50 plate-reader (Tecan).

TNF-α and IL-17A were quantified by sandwich ELISA with respectively TNF-α mouse uncoated ELISA kit (Invitrogen, Cat. No 88-7324-88) and IL-17A (homodimer) mouse uncoated ELISA kit (Invitrogen, Cat. No 88-7371-88) following the recommended manufacturer’s procedures and optical density was read at 450 nm on an Infinite F50 plate-reader (Tecan).

The respective dynamic ELISA assay ranges are 20–5000 pg/mL for IFN-γ; 8–1000 pg/mL for TNF-α and 4–500 pg/mL for IL-17A and appropriate serial dilutions of the samples were tested to quantify these three different cytokines.

ELISA results were analysed by converting measured absorbance to concentrations (pg/ml) by linear regression of the optical densities measured for the diluted standards using the Magellan for F50 software (Tecan).

### ELISA on samples form intranasally *Mtb* infected and subcutaneously BCG vaccinated mice

*Mtb* infected splenocytes (300,000 cells/well) were incubated with single recombinant proteins or mitogens (5 µg/ml) at 37°, 5% CO_2_. After 6 days of stimulation, the supernatants were filtered through 0.2 µM filter plates (Corning, U.S.A.). The IFN-γ ELISA (BD Bioscience, U.S.A, capture antibody cat no 551216 and biotin labelled detection antibody cat no 554410) was performed on the filtered supernatants according to manufacturer’s instructions. Absorbance (OD450) was determined and converted to concentrations using a standard curve by the Microplate Manager software version 5.2.1 (Biorad Laboratories, The Netherlands) (Supplementary Data 2).

### Data analysis

For each cytokine, within each pool of mice (Supplementary Data 1) or single mouse (Supplementary Data 2), the background-value, i.e., the value determined for unstimulated samples, was subtracted from the value determined for each antigen or mitogen. Data obtained at the later stage are pooled for analysis (9–12 weeks). The Kruskal–Wallis test was performed to assess the difference between the responses found in uninfected mice versus infected mice at different time points or versus BCG immunised mice for each antigen using IBM SPSS Statistics 26 (Figs 1b and 2b, Supplementary Data 1 and 2). Due to the high number of comparisons included in the study, we performed a two-sided Mann-Whitney test with an FDR (Benjamini-Hochberg) correction (results reported in the Supplementary Data 1 and 2). However, significant responses to positive controls or to previously described antigens (such as ESAT6/CFP10) were lost after correction (with an FDR cut-off set at 0.1), questioning whether this more stringent approach could lead to the exclusion of interesting candidates at this early stage of discovery. Thus, results of both unadjusted and adjusted tests are reported. In addition, for the first set of experiments, we analysed the responses to specific antigens on the basis of known classes ((i) naïve mice vs. mice killed at early or late point after *Mtb* infection; (II) mouse strains; (iii) organs; (iv) cytokines) using a supervised partial least squares-discriminant analysis (PLS-DA) method (Supplementary Fig. 2a) and a principal component analysis (PCA) (Supplementary Fig. 2b). The median responses to each specific antigen (Supplementary Data 1) were explored and analysed with ‘mixOmics’ (package version 6.15, http://www.mixOmics.org) in R (version 4.0.3) after log_10_ transformation. Three-component models explained the highest proportion of variance in the PCA as well as lowest balanced error rate (BER) in the PLSA-DA (Supplementary Data 1). For each of the categories investigated through PLSA-DA, we obtained Variable Importance in Projection (VIP) values which are a quantitative estimation of the discriminatory power of each individual antigen in a specific model (Supplementary Data 1 and Fig. 1c). The VIP values vary in a fixed range since the sum of squared VIP scores for all variables sum to the number of variables^49^. Therefore, antigens with a VIP value larger than 1 (i.e., larger than the average of squared VIP values) were considered to have an above average influence on the model^49^.

The Kruskal–Wallis test with Dunn’s multiple test correction was applied in Graphpad Prism (version 8) to analyse cytokine responses measured for all the 27 *Mtb* antigens across organs and timepoints (Supplementary Fig. 1).

Of note, in the second experiment, the IFN-γ response to HPV16E6, used as negative control, was higher in mice after BCG or *Mtb* infection than in the uninfected group. This response could be due to a non-specific immune activation profile. Therefore, we considered responses to be significant and specific only when the median IFN-γ production exceeded twice the IFN-γ production found in response to the negative control in BCG immunised and *Mtb* infected mice.

### Reporting summary

Further information on research design is available in the Nature Research Reporting Summary linked to this article.

## Supplementary information

Supplementary Information

Supplementary Data 1

Supplementary Data 2

Reporting Summary

## Data Availability

All data generated or analysed during this study are included in this published article and its supplementary information files.

## Code availability

The code used in this study was obtained from the ‘mixOmics’ (package version 6.15, http://www.mixOmics.org) for R (version 4.0.3).
