# Supplementary material for: In-vivo expressed Mycobacterium tuberculosis antigens recognised in three mouse strains after infection and BCG vaccination
Source: NPJ Vaccines. 2021 Jun 3;6:81. doi: 10.1038/s41541-021-00343-2 (PMC8175414; doi:10.1038/s41541-021-00343-2)
Supplement: Supplementary file 1 — Supplementary Information [file 41541_2021_343_MOESM1_ESM.pdf]

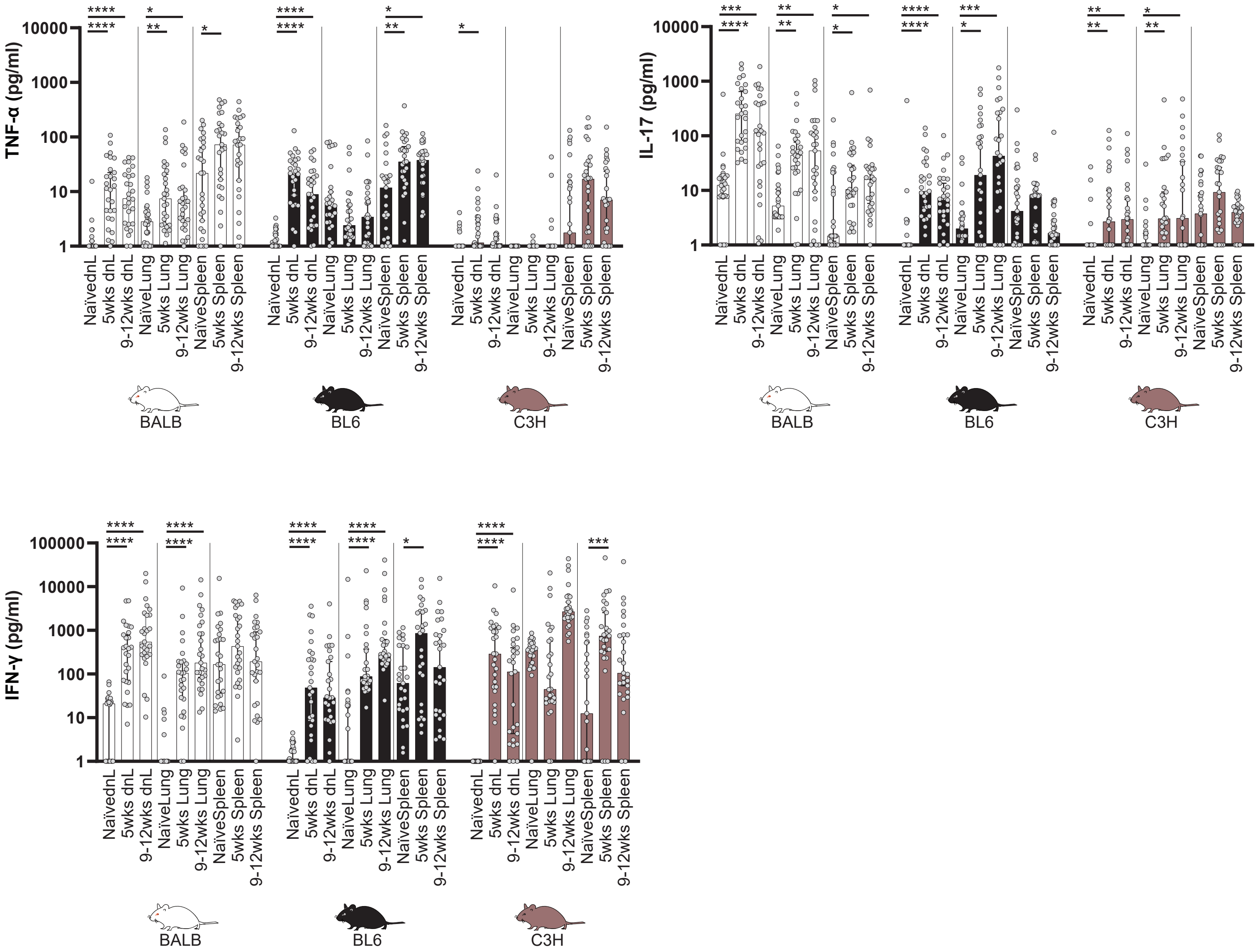

Supplementary Figure 1. Cumulative response against all *Mtb* antigens evaluated in Figure 1.
The bar graphs show the TNF-α, IL-17 and IFN-γ production after *Mtb* antigen stimulation of cells from mediastinal lymph nodes (medLN), lungs, and spleens from naïve or *Mtb* infected (5 or 9-12 weeks(wks)) C57BL/6 (BL6), BALB/c (BALB) and C3HeB/FeJ (C3H). Dots represent the response against a single antigen, while bars depict the median response against all antigens. (*: p<0.05, **: p<0.01, ***: p<0.001, ****: p<0.0001, Kruskal-Wallis test with Dunn's multiple test correction).

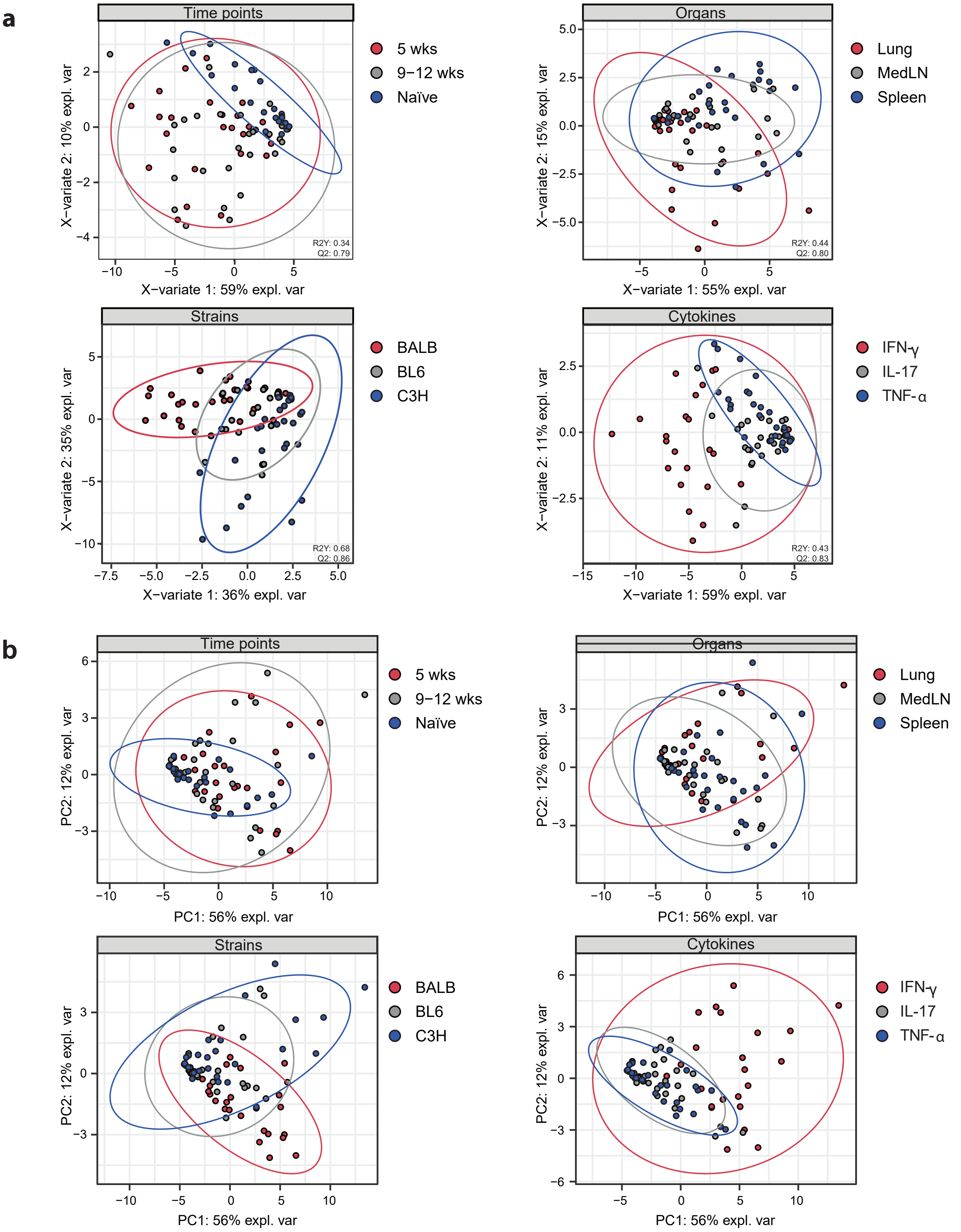

Supplementary Figure 2. PLS-DA and PCA models – related to Figure 1C.
(a) PLS-DA and (a) PCA score plots show the separation between antigens on the basis of known classes: (i) time points (i.e., naïve mice vs. mice sacrificed at early or late point after *Mtb* infection) (upper left); (II) mouse strains (i.e., C57BL/6 (BL6), BALB/c (BALB) and C3HeB/FeJ (C3H) mice) (lower left); (iii) organs (i.e., lungs, mediastinal lymph nodes or spleens) (upper right); (iv) cytokines (i.e., IFN-γ, TNF-α and IL-17) (lower right). Colored ovals represent PLS-DA and PCA protein clusters. Dots represent antigens and color code marks the groups included in each of the models as described in the figure.

medLN BL6

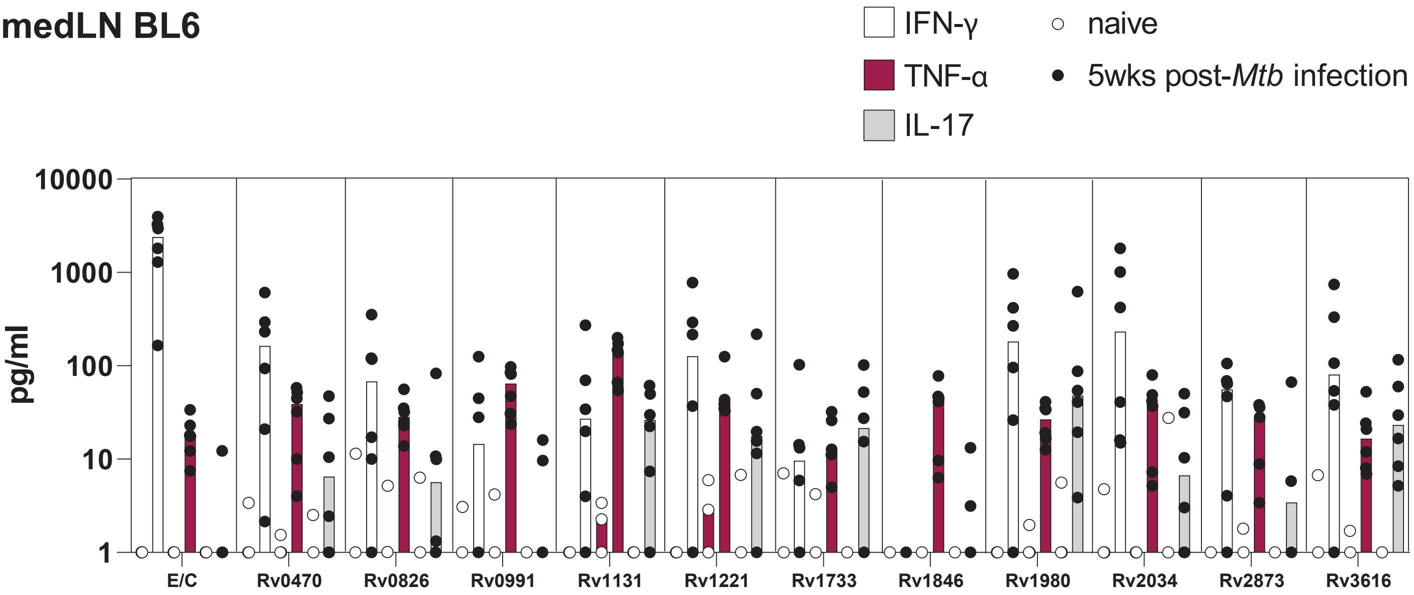

Supplementary Figure 3. Cytokine profiles in response to antigen stimulation in the mediastinal lymph nodes (medLN) of C57BL/6 (BL6) mice – related to Figure 1B.

Dot plot graphs depict the TNF-α, IL-17 and IFN-γ production after *Mtb* antigen stimulation of cells from mediastinal lymph nodes (medLN) from C57BL/6 (BL6). White and black solid dots depict the cytokine responses in the naïve and the *Mtb* infected (5 weeks (wks) post-infection) groups, respectively. White, purple and grey bars depict the median IFN-γ, TNF-α and IL-17 concentration, respectively. This figure contains only antigens that, after FDR correction, induced a significant increase of TNF-α but not IFN-γ or IL-17 in the medLN. ESAT6/CFP10 (E/C) was included for comparison.

Supplementary Table 1. Methodologies used in this multicenter study

	Figure 1	Figure 2
**Number of screens**	Three	One
**Mouse strains**	C57BL/6, BALB/c and C3HeB/FeJ mice (JAX stock #000658)	C3HeB/FeJ (JAX stock #000658)
**Route of infection**	Intratracheal	Intranasally
**Infection with *Mtb***	$1x10^4$ CFU of virulent and luminescent *Mtb* H37Rv	$1x10^5$ CFU live *Mtb* strain H37Rv
**BCG immunization**	No	Yes
**Time points evaluated after infection**	Multiple	Single
**Type of cells stimulated *in vitro***	Pools of splenocytes, lung and mediastinal lymph nodes cells isolated from 4-13 organs/experimental group	Splenocytes from single mouse
**Days of *in vitro* stimulation**	Three	Six
**Number of splenocytes stimulated per well***	720000	300000
**Controls**	ConA and PWM	ConA, PPD, bead disrupted BCG, *Mtb* lysate and HPV16E6

*details for lung and mediastinal lymph nodes cells in the Supplementary Data 1; *Mtb*: *Mycobacterium tuberculosis*; CFU: colony forming units; ConA: Concanavalin A; PWM: Pokeweed Mitogen; BCG: Bacillus Calmette–Guérin; PPD: purified protein derivative; HPV16E6: human papillomavirus recombinant protein.

LEGENDS TO SUPPLEMENTARY DATA

Supplementary Data 1. Raw and processed data underlying Figure 1.
Supplementary Data 2. Raw and processed data underlying Figure 2.
